# Influence of serum adiponectin on baseline albuminuria and subsequent decline in eGFR in African Americans: The Jackson Heart Study

**DOI:** 10.1371/journal.pone.0335611

**Published:** 2025-11-18

**Authors:** Mohit Agarwal, Prakrati C. Acharya, Aditi Kumar, Norah Sohail, Luis A. Juncos, Bessie A. Young

**Affiliations:** 1 Division of Nephrology, Banner – University Medical Center, Phoenix, Arizona, United States of America; 2 University of Arizona, Phoenix, AZ, USA; 3 Division of Nephrology, Carl T Hayden VA Medical Center, Phoenix, Arizona, United States of America; 4 Division of Endocrinology, Mayo Clinic, Scottsdale, Arizona, United States of America; 5 Arizona State University, Phoenix, Arizona, United States of America; 6 Director of Global Services in Critical Care, Fresenius Medical Center, Little Rock, Arkansas, United States of America; 7 Division of Nephrology, University of Washington, Seattle, Washington, United States of America; Medical School, University of Zagreb, CROATIA

## Abstract

**Purpose:**

Few studies to date have examined the relationship of adiponectin with kidney disease in an exclusively African American (AA) population. The purpose of this study was to determine whether serum adiponectin was an independent predictor of the presence of albuminuria or decline in estimated glomerular filtration rate (eGFR) in AA.

**Methods:**

The study included 5,301 AA participants enrolled in the Jackson Heart Study (JHS), Exam 1 and 3 (2000–2013). The primary outcome measure was the decline in eGFR. Cross sectional analyses were used to assess the relationship between albuminuria and serum adiponectin. Longitudinal analyses were used to assess the relationship between eGFR decline and adiponectin.

**Results:**

Nearly 2/3 of subjects were women (63.5%) and about half (52%) of the participants were middle aged (45–65 years). Mean eGFR was 94.15 (±SD 21.98) mL/min/1.73 m^2^ and 6.30% of subjects had eGFR < 60 mL/min/1.73 m^2^ Mean serum adiponectin levels were 5408.69 ng/mL (±SD 4,280.32 ng/mL) and women had higher serum adiponectin levels 6139.14 ng/mL (±SD 4553.57 ng/mL) than men 4148.91 ng/mL (±SD 3414.43 ng/mL) (p < 0.001). Analyses revealed positive correlation between adiponectin levels and albuminuria (P = 0.001), an association significant only in men.

During a median duration of follow up of approximately 8 years, a higher baseline adiponectin was associated with a subsequent higher decline in eGFR. Due to the skewed distribution and to facilitate the interpretation of results, we transformed baseline Adiponectin values to log base 2. So a unit higher baseline Log based 2 Adiponectin indicates a level two times the lower value. And an African American man with twice the baseline value of adiponectin had a risk of 2.22 mL/min/1.73 m^2^ higher subsequent decline in GFR (95% CI: −3.72 to −0.73; P = 0.003) over the study period. This effect was not seen in women. On further subgroup analysis of those with a rapid kidney function decline (eGFR rate loss >3.5 mL/min/year), the correlation between adiponectin and eGFR became more prominent (4.7 mL/min/1.73 m^2^ decline in eGFR per 10 years) (P = 0.006). No association was seen between baseline adiponectin and progressive eGFR decline in subgroups of men without rapid kidney function decline.

**Conclusion:**

In AA men, elevated adiponectin level at baseline is an independent predictor of albuminuria and subsequent decline in eGFR.

## Introduction

Obesity is a well-recognized risk factor for chronic kidney disease (CKD) [1–5] and end stage kidney disease (ESKD), as well [3].Kidney biopsies from individuals who are obese have revealed a distinct pathological picture characterized by glomerulomegaly and patchy foot process fusion, which is different from that of idiopathic focal segmental glomerulosclerosis (FSGS) [6]. Adipose tissue has been recognized as an important endocrine organ responsible for production of key homeostatic substances and hormones such as adiponectin [7–9]. Limited information is known regarding the association of adiponectin and kidney function or kidney function decline.

Adiponectin is a 244-amino acid, 30 kDa anti-inflammatory and insulin sensitizing protein hormone almost exclusively secreted by the white adipose tissue. It is the most abundant adipokine in the serum [10], and multiple studies have linked low levels of adiponectin with increased risk of endothelial dysfunction, hypertension, obesity and insulin resistance [11] Conversely, a protective role of adiponectin has been implicated in cardiovascular and bronchial inflammatory states [12–17]. In several animal and human studies, adiponectin levels have been inversely linked to albuminuria and glomerulosclerosis. For example, in adiponectin null mouse, Sharma et al demonstrated amelioration of podocyte abnormalities and albuminuria by adiponectin supplementation [18]. In humans, hypoadiponectinemia has been found to be associated with microalbuminuria in hypertensive patients [19] as well as obese non-diabetic Japanese patients [20]. Hypoadiponectinemia has also been linked to the progression of albuminuria in type 2 diabetes patients [21]. Also, higher adiponectin levels have been associated with reduced odds of kidney dysfunction in type 2 diabetics [22]. Interestingly, in contrast to the above studies, high adiponectin levels have also been found to be associated with more severe proteinuria in CKD patients [23], earlier onset of ESRD, and increased mortality in type 1 diabetics [24–26]. This paradoxical response can be interpreted either as compensatory increase in adiponectin levels or as resistance to adiponectin.

Few studies have examined the longitudinal relationship of adiponectin with kidney disease in an exclusively African American population and as a result its role in the pathogenesis of kidney diseases in this population remains poorly defined [27]. The present study is focused on adiponectin’s association with kidney function. This study question is particularly relevant clinically as African American individuals tend to have a higher burden of CKD. Moreover, even though there are some cross-sectional data suggesting that the adiponectin levels are higher in CKD compared to non-CKD populations [28], it is unclear if this is a true association or just a reflection of reduced adiponectin clearance in the setting of failing kidneys [29,30]. Most of the previously published studies were limited by small sample sizes and cross-sectional design. Therefore, in the present study we attempted to explore the longitudinal relationship between adiponectin and kidney function in a large racially homogeneous (African American) cohort. In addition, due to known sex difference of adiponectin in terms of serum levels and circulating isoforms [31], we have analyzed circulating adiponectin in relation to albuminuria and eGFR stratified by sex.

## Materials and methods

### Study population

The Jackson heart study (JHS) is the one of the largest prospective cohort studies designed to evaluate causes of cardiovascular disease from an exclusively African American population. Design and methods of the JHS have been described elsewhere [32]. Briefly, in this single site longitudinal population-based study, 5,301 participants were recruited between 2000 and 2004 (Exam 1) from the Jackson, Mississippi, metropolitan area (Hinds, Madison, and Rankin counties). The data was accessed on July 22,2015. Participants were aged 20 years and older when they enrolled in the study. Exam 2 collected data from 2005–2008, while Exam 3 began in 2009 and ended in 2013. Institutional review boards of University of Mississippi Medical Center, Jackson State University, and Tougaloo College have approved the study. Written informed consent was provided by each participant.

### Demographic variables and data collection

Study subjects enrolled in the Jackson Heart Study (JHS) from 2000–2013 included 5,301 adult African American participants. In addition to serum adiponectin, urine albumin to creatinine ration (ACR), and eGFR, we retrieved the following data: age (defined in years), sex (male/female), smoking, hypertension (blood pressure 140/90 mm Hg or use of blood pressure medications), diabetes (defined as having blood glucose level greater than 126 mg/dL, hemoglobinA1c level > 6.5%, or receiving insulin or other hypoglycemic agents), high density lipoprotein (HDL) (mg/dL) and body mass index (BMI) (kilograms per meter squared), as previous research [17,21,33] has documented that these effects may affect eGFR and/or adiponectin levels, thus confounding the effect of serum adiponectin on eGFR.

### Laboratory measurements

#### University hospital and clinics laboratory at the University of Mississippi Medical Center (UMMC).

Urine albumin was measured on a Dade-Behring BN II nephelometer (Newark, Delaware). Serum and urine creatinine were measured on a Vtros 950, Ortho-Clinical Diagnostics analyzer (Raritan, New Jersey) [34,35]. Creatinine values were biochemically calibrated to Cleveland Clinic-equivalent Minnesota Beckman CX3 assay (Beckman-Coulter Inc, Fullteron, CA) for analysis purposes and eGFR was calculated via the Chronic Kidney Disease Epidemiology Collaboration (CKD-EPI) equation 2009 [35,36].

#### University of minnesota laboratory.

All adiponectin measurements were collected and measured during Exam 1 from 2000 to 2004 (baseline). Venous blood samples were withdrawn from each subject at baseline examination after more than 8h of fasting. Vials of serum were stored at the JHS central laboratory in Minneapolis, MN, USA at −80 °C until assayed. Adiponectin level in the serum was measured as total adiponectin by an ELISA system (R&D Systems; Minneapolis, MN, USA) [37]. It is to be noted that no biological degradation has been described with stored specimens [38]. HDL-cholesterol was measured using a Roche COBAS Fara analyzer (Indianapolis, Indiana) [34]

#### End-point assessment.

The primary outcome measure was the decline in eGFR over the course of the study (time points at Exam 1 (2000–2004) and Exam 3 (2009–2013)). Serum creatinine was measured using the Vitros Ortho-Clinical Diagnostics Analyzer (Raritan, NJ) [34]. Serum creatinine was re-measured in 2006 for 206 participants using the enzymatic method on a Roche Chemistry analyzer (Roche Diagnostics Corp, Indianapolis IN). We estimated eGFR from serum-calibrated creatinine using the 4-variable CKD epidemiology (CKD-EPI) equation, [39] which was derived from a series of pooled cohorts that used the iothalomate clearance as the criterion standard. Rapid kidney function decline (rapid decline) was defined as an annual loss of 3.5 mL/min/1.73m^2^ or more [40,41]. The trajectory of rapid decline was based on 2 time points available from serum creatinine tests available from Exams 1 (2000–2004) and 3 (2009–2013). Albuminuria was defined as a urinary-albumin-to-urinary-creatinine ratio (ACR) ≥30 mg/g. Spot urine specimen was used to calculate ACR.

### Statistical analysis

All statistical analysis was performed in STATA (STATA, version 14.2, College Station, Texas). Descriptive statistics were used to describe the baseline characteristics of the study population. Values are given as means ± standard deviation (SD) or absolute numbers (percentages). Differences between sexes were analyzed using two sample mean comparison tests. The chi-squared test was used to compare categorical variables. Continuous variables were examined for normality and skewness. Urine ACR, and serum adiponectin were log transformed (with base 2) before linear regression analyses were conducted.

We conducted both cross-sectional and longitudinal analyses. Cross sectional analyses were used to assess the hypothesized relationship between urine ACR and serum adiponectin at baseline while adjusting for potential covariates. We conducted the analysis of study data using regression models. We used linear models. A random effect model was used to account for internal correlation between repeated subject observations, using Stata command xtreg for random effects analysis. The first model was unadjusted. Subsequently, the confounding influence of GFR, age, smoking, hypertension, HDL, diabetes, and BMI was tested. Longitudinal analyses were used to assess the hypothesized relationship between baseline serum adiponectin and the decline in eGFR over the course of the study, while adjusting for potential covariates. Using the “xtreg’ command of the statistical software package STATA, with time-points at Exam 1 (2000–2004) and Exam 3 (2009–2013), two-level multilevel linear regression models were fitted to estimate the contribution of serum adiponectin on the decline of eGFR. This approach enabled us to account for the correlated data structure across the two timepoints. Analyses were stratified by sex due to known sex differences in total adiponectin levels and also in the levels of various isoforms [31]. The first model was unadjusted. Subsequently, the confounding influence of GFR, age, smoking, hypertension, HDL, diabetes, BMI, and urine ACR was tested. Linear change in eGFR was assessed by multivariable linear regression as the change in eGFR from Exam 1 (2000–2004) to Exam 3 (2009–2013) over time.

Subjects were then divided into two subgroups depending upon whether they experienced a rapid decline in kidney function (defined as a decline in eGFR loss of greater than 3.5 mL/min/year [40]) or not. Sex stratified longitudinal analyses were again repeated to assess the hypothesized relationship between baseline serum adiponectin and the decline in eGFR over the course of the study, while adjusting for potential covariates.

## Results

Of the 5,301 subjects, baseline eGFR and serum adiponectin were available for 5,210 and 5,106 subjects respectively (Flow Chart 1). Baseline Exam 1 and Exam 3 characteristics subdivided by sex are summarized in the Tables 1–4. 64 Percent were women and 52% of the participants were middle aged (45–65 yrs). Mean eGFR was 94.15 (±SD 21.98) mL/min/1.73 m^2^ and 6.30% of subjects had an estimated GFR of less than 60 mL/min/1.73 m^2^. Mean serum adiponectin level was 5,408.69 ng/mL (±SD 4280.32 ng/mL) and median serum adiponectin levels were 4247.40 ng/mL (min 351.91, max 62861.51, IQR 4105.07). Sex differences were noted with women having higher levels. Mean serum adiponectin level for men was 4,148.91 ng/mL [±SD 3414.43 ng/mL], Median serum adiponectin level for men was 3143.67 ng/mL[min 370.98, max 39697.18, IQR 3054.46]. Mean serum adiponectin level for women was 6,139.14 ng/mL (±SD 4553.57 ng/mL). Median serum adiponectin level for women was 4945.30 ng/mL [min 351.91, max 62861.51, IQR 4407.41] [11].

**Table 1:** Baseline Exam 1 characteristics stratified by sex. Mean values shown with SD in parentheses.

	(1)	(2)	(3)	(4)
	Full sample	Men	Women	P Value
	mean	mean	mean	p
Age [years]	55.36 (12.85)	54.58 (12.96)	55.82 (12.76)	0.001
BMI [Kg/m^2^]	31.75 (7.24)	29.86 (6.14)	32.84 (7.59)	<0.001
*Current Smoker, %	13%	18%	10%	<0.001
*Hypertension, %	60%	58%	62%	0.004
*Diabetes, %	22%	20%	23%	0.031
HDL [mg/dL]	51.77 (14.64)	45.93 (12.67)	55.10 (14.64)	<0.001
Serum Adiponectin [ng/mL]	5408.69 (4280.32)	4148.91 (3414.43)	6139.14 (4553.57)	<0.001
eGFR [mL/min/1.73 m^2^]	94.15 (21.98)	92.89 (20.52)	94.88 (22.76)	0.002
*N*	5301	1934	3367	

*Non-Continuous Variables. Percentage.

BMI – Body Mass index, HDL – High density lipoprotein, eGFR – Estimated glomerular filtration rate.

**Table 2:** Exam 3 characteristics stratified by sex. Mean values shown with SD in parentheses.

	(1)	(2)	(3)	(4)
	Full sample	Men	Women	P Value
	mean	mean	mean	p
Age [years]	62.33 (12.09)	61.20 (11.95)	62.98 (12.13)	<0.001
BMI [Kg/m^2^]	32.15 (7.23)	30.38 (6.27)	33.16 (7.54)	<0.001
*Hypertension, %	26%	26%	25%	0.92
*Diabetes, %	31%	30%	32%	0.19
HDL [mg/dL]	57.95 (16.06)	52.33 (14.74)	61.12(15.90)	<0.001
eGFR [mL/min/1.73 m^2^]	85.71(23.23)	84.65 (21.80)	86.32 (24.00)	0.04
*N*	3697	1347	2350	

*Non-Continuous Variables. Percentage.

BMI – Body Mass index, HDL – High density lipoprotein, eGFR – Estimated glomerular filtration rate.

**Table 3:** Baseline Exam 1 characteristics of subjects with available Adiponectin values (n = 5106). Mean values shown with SD in parentheses.

	Mean
Age [years]	55.19 (12.82)
BMI [Kg/m^2^]	31.75 (7.23)
*Current Smoker, %	13%
*Hypertension, %	60%
*Diabetes, %	22%
HDL [mg/dL]	51.74 (14.63)
Serum Adiponectin [ng/mL]	5408.69 (4280.32)
eGFR [mL/min/1.73 m^2^]	94.41 (21.77)
Males	37%
*N*	5106

**Table 4:** Baseline Exam 1 characteristics of subjects with available ACR values (n = 2554). Mean values shown with SD in parentheses.

	Mean
Age [years]	52.52 (13.04)
BMI [Kg/m^2^]	31.59 (7.15)
*Current Smoker, %	12%
*Hypertension, %	57%
*Diabetes, %	19%
HDL [mg/dL]	49.80(13.42)
Serum Adiponectin [ng/mL]	5434.36 (4526.19)
eGFR [mL/min/1.73 m^2^]	97.10 (21.40)
Males	40%
*N*	2554

**Fig 1:**
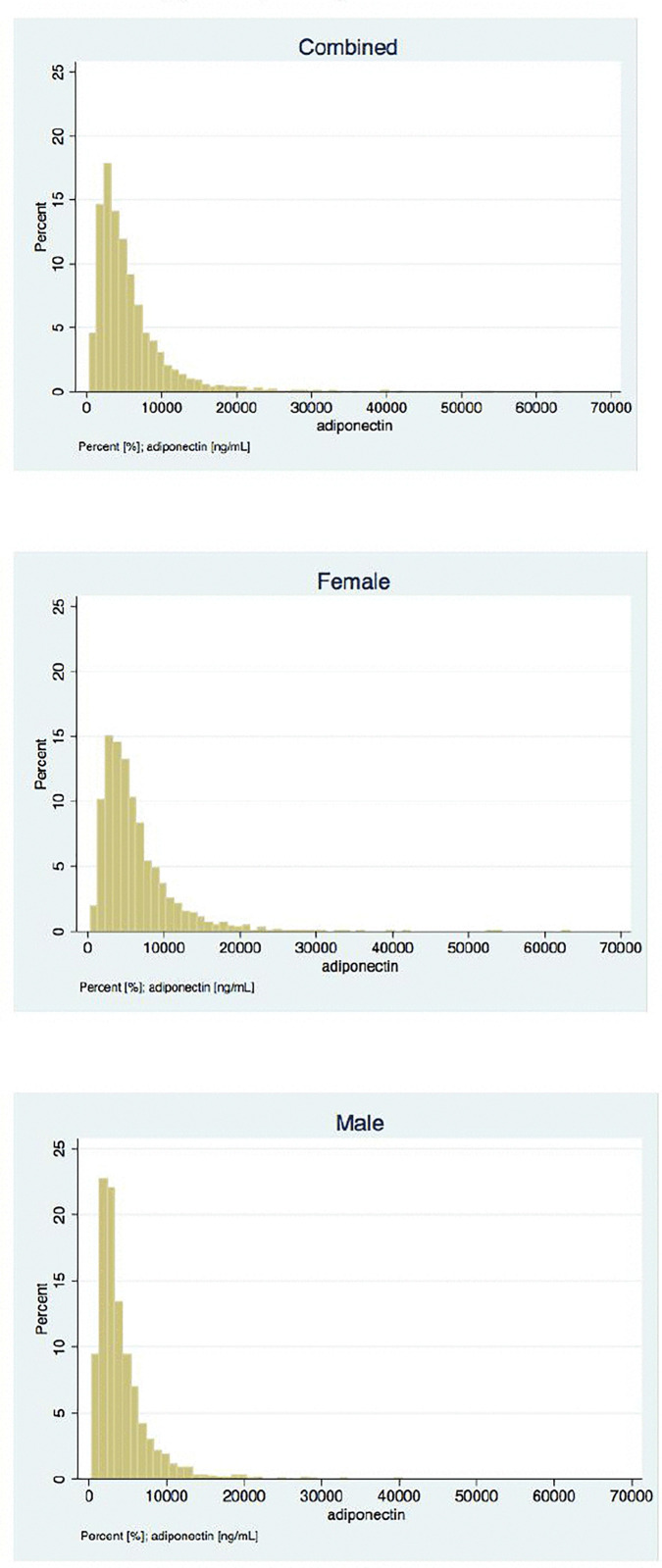
Relationship between albuminuria and serum adiponectin at baseline.

Of the 5,301 subjects, baseline urine ACR data were available for 2,554 subjects. Urine ACR data were skewed and required log transformation before regression analyses were conducted. After adjusting for potential confounders (GFR, age, smoking, hypertension, HDL, diabetes, and BMI) a direct association between albuminuria and adiponectin level at baseline was noted. Interestingly, after stratification by sex this effect was found to be significant only in men (Table 5 and Fig 1).

**Table 5:** Association between albuminuria and Adiponectin level at baseline adjusted for cofactors.

ACR [log2] [mg/g]**	Coef.	SE	t	P	95% Conf. Interval	
N = 2,257	Both sexes					
Adiponectin [log2] [Serum ng/mL]**	0.12	0.04	2.93	0.003	0.039	0.19
N = 1,361	Females					
Adiponectin [log2] [Serum ng/mL]**	0.04	0.05	0.83	0.407	0.06	0.15
N = 896	Males					
Adiponectin [log2] [Serum ng/mL]**	0.20	0.06	3.36	0.001*	0.09	0.33

*Direct relationship between Adiponectin Level and Albuminuria was seen only in Males.

**Baseline (exam # 1) Urine Albumin Creatinine Ratio (ACR) and Serum Adiponectin had to be log transformed to adjust for skewness of the distribution.

-Significant p-value for a characteristic means that characteristics were significantly different between sexes.

-Adjusted for GFR, age, smoking, hypertension, HDL, Diabetes, and BMI; combined and then for women and men separately.

Definitions: ACR = albumin to creatinine ratio.

The median duration between baseline Exam 1 (2000–2004) and follow up Exam 3 (2009–2013) was 2,928 days (≈8.02 years). Of the 3,815 subjects, eGFR was available for 3,758 subjects. In men, at Exam 3 (median 8.02 years after exam 1), a higher baseline adiponectin at the time of Exam 1 was associated with a subsequent higher decline in eGFR. Due to the skewed distribution and to facilitate the interpretation of results, we transformed baseline Adiponectin values to log base 2. So a unit higher baseline Log base 2 Adiponectin indicates a level two times the lower value. And an African American man with twice the baseline value of adiponectin had a risk of 2.22 mL/min/1.73 m2 higher decline in GFR (95% CI: −3.72 to −0.73; P = 0.003) over the study period. This effect was not seen in women (Table 6).

**Table 6:** Association between decline in eGFR from baseline to follow up and Serum Adiponectin level at baseline.

eGFR	Coef.	SE.	t	P	95% Conf. Interval	
Females (Unadjusted)						
Adiponectin [log2] [Serum ng/mL]**	-.614	0.35	-1.75	0.079	-1.23	0.07
Males (Unadjusted)						
Adiponectin [log2] [Serum ng/mL]**	-1.66	0.53	-3.18	0.001*	-2.69	-.638
Females (Adjusted for GFR, age, smoking, hypertension, HDL, Diabetes, and BMI)						
Adiponectin [log2] [Serum ng/mL]**	-.699	0.35	-1.96	0.051	-1.40	0.001
Males (Adjusted for GFR, age, smoking, hypertension, HDL, Diabetes, and BMI)						
Adiponectin [log2] [Serum ng/mL]**	-1.835	0.54	-3.38	0.001*	-2.89	-.770
Females (Adjusted for above and **urine albumin creatinine ratio (ACR))**						
Adiponectin [log2] [Serum ng/mL]**	-.648	0.50	-1.29	0.196	-1.63	.334
Males (Adjusted for above and **urine albumin creatinine ratio (ACR))**						
Adiponectin [log2] [Serum ng/mL]**	-2.22***	0.76	-2.92	0.003*	-3.71	-.732

*Direct relationship between decline in eGFR at exam # 3 (Median 8.02 years after exam # 1) and adiponectin level at baseline was seen only in Males.

**Baseline (exam # 1) serum adiponectin and urine albumin creatinine Ratio (ACR) had to be log transformed to adjust for skewness of the distribution.

***During a median duration of follow up of approximately 8 years, a higher baseline adiponectin was associated with a subsequent higher decline in eGFR. Due to the skewed distribution and to facilitate the interpretation of results, we transformed baseline Adiponectin values to log base 2. So a unit higher baseline Log based 2 Adiponectin indicates a level two times the lower value. And an African American man with twice the baseline value of adiponectin had a risk of 2.22 mL/min/1.73 m^2^ higher decline in GFR (95% CI: −3.72 to −0.73; P = 0.003) over the study period. This effect was not seen in women.

-Unadjusted; Adjusted for GFR, age, smoking, hypertension, HDL, diabetes, and BMI; Adjusted for aforementioned and urine albumin creatinine ratio (ACR); for women and men separately.

**Fig 2:**
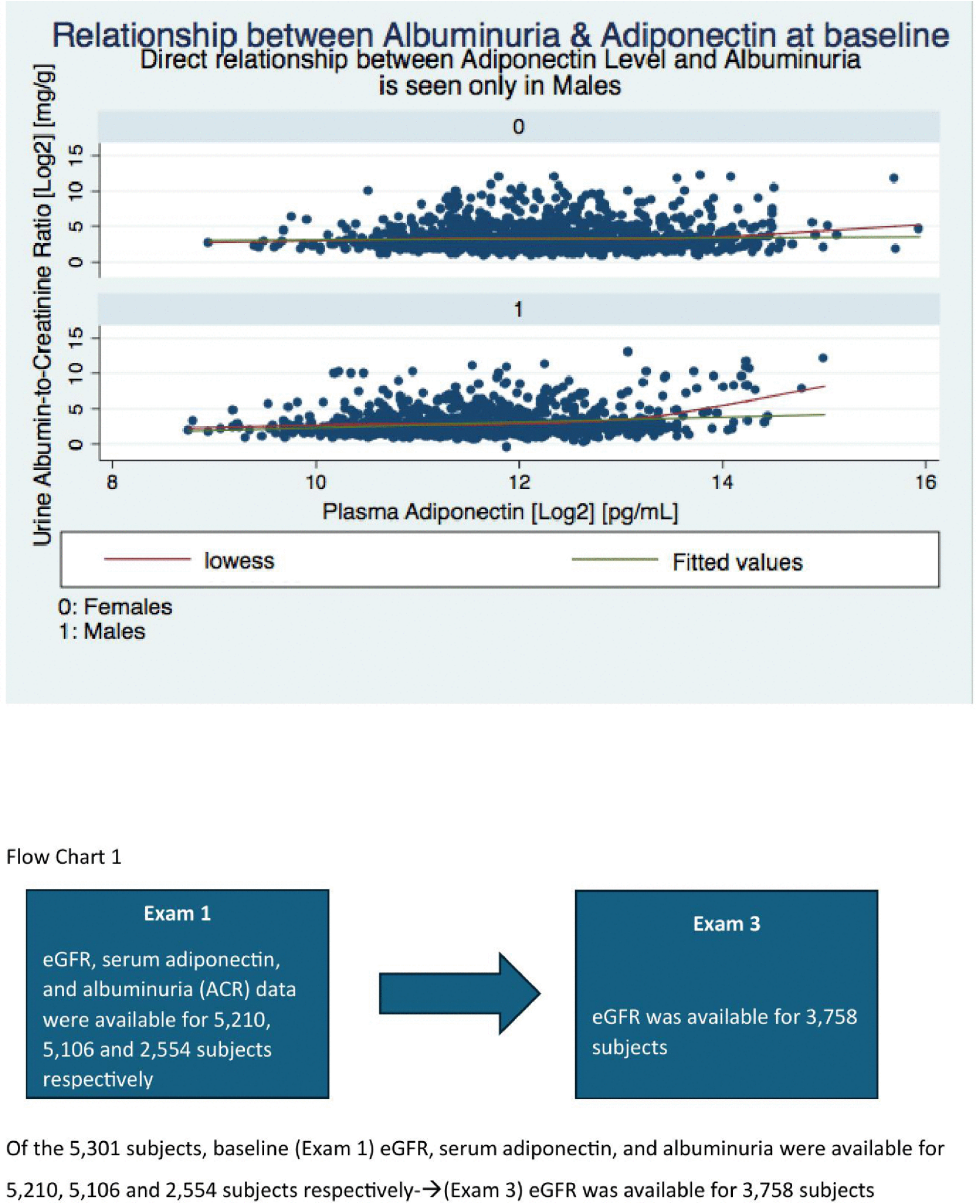
Percentage frequency distribution of Serum Adiponectin at baseline by sex.

We performed additional sub-group analysis to determine the differential effect of baseline adiponectin in groups stratified by the rapidity of kidney function decline and sex. Serum adiponectin data were skewed and required log transformation before linear regression analyses were conducted (Fig 2).

398 subjects had rapid kidney function decline (eGFR loss >3.5 mL/min/year). In the sub-group of men who experienced this rapid kidney function decline, each unit higher baseline Log base 2 Adiponectin (indicating a level two times the lower value), was found to be associated with a subsequent 4.7 mL/min/1.73 m^2^ decline (per 10 years) in eGFR (P = 0.006). Once again, this effect was not seen in women, or in subjects who did not experience a rapid decline in renal function (Table 7).

**Table 7:** Association between decline in eGFR (per 10 years) and Serum Adiponectin level at baseline.

Subgroup analysis: Patients who experienced a rapid decline in renal function (estimated glomerular filtration rate loss >3.5 mL/min/year; n = 398)												
**eGFR decline per 10 years**	**Coef.**		**SE**		**t**		**P > t**		**95% Conf. Interval**			
Females (Unadjusted)												
Adiponectin [log2] [Serum ng/mL]**	1.08		1.05		1.03		0.30		−.995		3.17	
Males (Unadjusted)												
Adiponectin [log2] [Serum ng/mL]**	2.52		1.52		1.65		0.102		−.50		5.54	
Females (Adjusted for GFR, age, smoking, hypertension, HDL, Diabetes, and BMI)												
Adiponectin [log2] [Serum ng/mL]**	1.68		1.24		1.35		0.177		−.76		4.13	
Males (Adjusted for GFR, age, smoking, hypertension, HDL, Diabetes, and BMI)												
Adiponectin [log2] [Serum ng/mL]**	4.76		1.69		2.80		0.006*		1.39		8.12	
Patients who did not experience a rapid decline in renal function (estimated glomerular filtration rate loss < 3.5 mL/min/year)												
**eGFR decline per 10 years**		**Coef.**		**SE**		**t**		**P > t**		**95% Conf. Interval**		
Females (Unadjusted)												
Adiponectin [log2] [Serum ng/mL]**		0.598		0.344		1.74		0.082		−.077		1.274
Males (Unadjusted)												
Adiponectin [log2] [Serum ng/mL]**		0.895		0.421		2.13		0.034		0.069		1.722
Females (Adjusted for GFR, age, smoking, hypertension, HDL, Diabetes, and BMI)												
Adiponectin [log2] [Serum ng/mL]**		0.108		0.390		0.28		0.781		−.657		0.873
Males (Adjusted for GFR, age, smoking, hypertension, HDL, Diabetes, and BMI)												
Adiponectin [log2] [Serum ng/mL]**		0.718		0.478		1.50		0.133		−.220		1.658

*Direct relationship between Adiponectin Level and eGFR was not seen in females, or in subjects who did not experience a rapid decline in renal function.

**Baseline (exam # 1) serum adiponectin had to be log transformed to adjust for skewness of the distribution.

-Unadjusted; Adjusted for GFR, age, smoking, hypertension, HDL, diabetes, and BMI; for women and men separately.

## Discussion

Adiponectin levels at baseline were found to be independently associated with albuminuria at baseline in men, an effect not evident in women (Table 5). Similarly, in our longitudinal analysis, elevated adiponectin level at baseline was found to be an independent predictor of subsequent decline in eGFR in men (2.22 mL/min/1.73 m^2^ over a period of 8 years per unit higher baseline Log base 2 adiponectin level (indicating a level two times the lower value). This effect was not seen in women (Table 6).

In subgroup analysis with rapid decline in eGFR (> 3.5 ml/min/year), we noticed that the correlation between adiponectin and eGFR became more prominent in men. After adjustment for GFR, age, smoking, hypertension, HDL, diabetes and BMI, each unit higher baseline Log base 2 adiponectin level (indicating a level two times the lower value). was found to be associated with a 4.7 mL/min/1.73 m^2^ decline in eGFR (per 10 years) (P = 0.006) Conversely, it lost its significance in subjects with relatively stable kidney function. This suggests that baseline adiponectin levels may be considered as a marker of poor prognosis in patients experiencing a rapid kidney function decline.

It is interesting to note that the relationship between adiponectin and chronic renal failure/ nephrotic syndrome is the opposite to that found in other diseases such as obesity and diabetes [42].

In contrast to metabolic syndrome, obesity, insulin resistance and coronary heart disease, where low adiponectin is seen.], the body’s response in Nephrotic Syndrome is to increase production and Adiponectin is markedly increased in patients with nephrotic syndrome [43].

Adiponectin functions by activating the adenosine monophosphate-activated protein kinase (AMPK) pathway via the AdipoR receptors, including AdipoR1 and AdipoR2 [44].

The AMPK pathway is a central regulator of cellular energy that is essential for maintaining normal renal physiology. As the kidney is a highly metabolic organ with enormous energy demands, proper AMPK function is critical for processes including electrolyte and water transport, cell survival, metabolism, and blood pressure regulation. Dysregulated AMPK is a feature of metabolic diseases associated with kidney injury, such as obesity and diabetes [45,46].

Activating AMPK has been shown to counteract the pathological features of these diseases. Pharmacological activation of AMPK in preclinical models has been shown to improve kidney function and correct underlying metabolic defects [47]. So it might be considered a counter-regulatory response to the inflammation and metabolic abnormalities characteristic of Nephrotic Syndrome perhaps in an attempt to protect the endothelium.

Our results are consistent with the report from Zoccali et al., who found that adiponectin levels were found to be markedly increased in nephrotic syndrome and there was a positive correlation between adiponectin levels and severity of proteinuria [43]. Our observations are also consistent with a report by Kollerits et al., [48], a study of non-diabetic White patients with CKD, in which elevated adiponectin levels in men (but not women) were found to be associated with a faster progression of CKD.

Adiponectin forms a wide range of multimeric species including trimeric, hexameric and the HMW (high-molecular-mass) oligomeric complex consisting of at least 18 protomers. HMW form is considered the most biologically active in mediating the insulin-sensitizing effects, whereas the central actions are attributed primarily to the hexameric and trimeric oligomers [49]. In regards to mechanisms of sex differences, it is known that women have different isoforms of adiponectin, which are higher in molecular weight due to their multimeric nature compared to the men who have a predominance of trimers which are much lower in molecular weight [31]. Variation in isoforms in women is linked to hormonal activity. Xu et.al, have shown that testosterone selectively reduces high molecular weight isoform secretion from adipocytes [50]. Therefore, the biologic effects of the adiponectin isoforms in the women might be different from that of the men. Also, estrogen has been shown to have effects on adiponectin levels. In a study by Leung et al total adiponectin and its high molecular weight (HMW) isoform were found to be negatively associated with estradiol and progesterone levels in women, suggesting that higher estrogen levels are linked to lower adiponectin levels [51]. A study by Martinez- Cigoni et al. found that in obese diabetic female rats, ovariectomy improved adiponectin secretion [52].

Theoretically, based on biological grounds, an inverse relationship is assumed between adiponectin levels and the clinical outcomes. However, this relationship appears to be quite complex in clinical settings and some factors associated with adverse outcomes such as inflammation, malnutrition, and low eGFR have the potential to attenuate, or even reverse the direction of this association [53,54].  Although it cannot be ruled out that the rise in adiponectin is causing a direct deleterious effect on podocytes in the men, available evidence suggests that this is less likely.

Adiponectin has been shown to reduce proteinuria and glomerulosclerosis in experimental models. Kidney disease is seen in adiponectin null mice in association with increased albuminuria and fusion of podocyte foot processes. Amelioration of these abnormalities, together with reduction in urinary and glomerular markers of oxidant stress has been noted with adiponectin supplementation [18]. In another mouse model, adiponectin has also been found to be renoprotective after podocyte injury [55]. It is therefore conceivable that the paradoxical association of elevated adiponectin levels with albuminuria in men and subsequent decline in eGFR seen in the present study is either a failed compensatory response or a reflection of adiponectin resistance. Conversely, little research is available to explain the sex differences in the above associations. It can be due to the fact that the women tend to have both a higher total and a higher proportion of high and medium molecular weight isoforms of adiponectin when compared with the men [31]; or perhaps it is due to some other unknown metabolic and phenotypic differences between the two sexes [48].

Some studies have attempted to define the optimal adiponectin cutoff values that differentiate most sharply between good and bad prognosis groups. For example, Kollerits et al., reported that while male patients with serum adiponectin levels above the cut-off point of 4 mcg/mL showed a significantly faster progression than those below this value, no significant difference was observed in women [48]. Unfortunately, this approach has the risk of artificial reduction in p values and overestimation of prognostic significance [56]. These cut-off points may be data and sex specific, thus making comparison between different studies difficult and can also introduce a statistical artifact [57] which cause spurious improvements in group-specific predictive power, without changes in the individual outcomes. Therefore, these cutoff points must be interpreted with caution. In general adult populations, typical plasma adiponectin levels range from approximately 2–30 µg/mL (2000–30000 nanogram/ml) depending on assay methods.

Studies have also looked at Urinary adiponectin as a new diagnostic index for chronic kidney disease due to diabetic nephropathy [58].

Clinically, the effect size of 2.22 mL/min/1.73 m^2^ eGFR loss per doubling of baseline serum adiponectin level appears small, but it may be highly relevant in the specific clinical context. Let’s considers two male subjects: one with adiponectin level of 1 mcg/mL (1,000 ng/mL), and the other one with 32 mcg/mL (32,000 ng/mL). Over a period of 8 years, the first subject’s eGFR will decline by only 2.22 mL/min/1.73 m^2^ (2.22 X 1) but second subject’s eGFR would decline by 11.1 mL/min/1.73 m^2^ (2.22 X 5), a highly worrisome rate of change. It should also be noted that serum adiponectin levels have a wide range in humans and the differential effect on eGFR can reach many multiples of 2.22.

### Strength and limitations

Our study has several strengths. The present study analyzed serum adiponectin as a continuous variable. This prevented the introduction of all the biases associated with arbitrary dichotomization and resulted in maximal statistical power. Additionally, all our study subjects were African Americans and from the same geographic region and thus, we avoided the profound confounding effect of race, and variation of local cultural background. Also, the current study population was the largest reported to date, thus allowing adjustment for multiple potential confounders. As we focused on longitudinal analysis of the decline of eGFR, we were also able to mitigate the potential confounding caused by the effect of preexisting CKD on baseline adiponectin levels and adiponectin clearance. This was particularly important, as very few prospective studies are available [25,48]. The limitations of our study are worth considering. Unfortunately we do not have data on cystatin C. We did not test for various isoforms of adiponectin in the study population, which might differ in their respective biological activity and actions. This potential interference was nevertheless somewhat ameliorated by the sex-stratified regression analyses. Secondly, as this was an epidemiological study, we could not test whether adiponectin has any deleterious effects on kidney physiology. This study does not prove a causal relationship between adiponectin levels and ACR or eGFR. Thirdly, adiponectin levels were measured only at baseline.

## Conclusion

Our study showed that in African American men, elevated adiponectin levels at baseline are independent predictors of albuminuria and subsequent decline in eGFR. These effects are not seen in the African American women.
